# Supplementary material for: The optical conductivity of few-layer black phosphorus by infrared spectroscopy
Source: Nat Commun. 2020 Apr 15;11:1847. doi: 10.1038/s41467-020-15699-7 (PMC7160205; doi:10.1038/s41467-020-15699-7)
Supplement: Supplementary file 1 — Supplementary Information [file 41467_2020_15699_MOESM1_ESM.docx]

**Supplementary Information for**

**The optical conductivity of few-layer black phosphorus by infrared spectroscopy**

Guowei Zhang *et al.*





**Supplementary Figure 1. Absorption and PL spectra of a 3L BP.** The spectra are measured for samples on PDMS substrate at room temperature. The Stokes shift is almost negligible, indicating good sample quality.





**Supplementary Figure 2. Reflectance contrast spectra of 1-4L MoS_2_.** The spectra are measured for samples on PDMS substrate at room temperature. A/B excitons correspond to direct-gap transitions at Κ point of the 2D Brillouin zone.


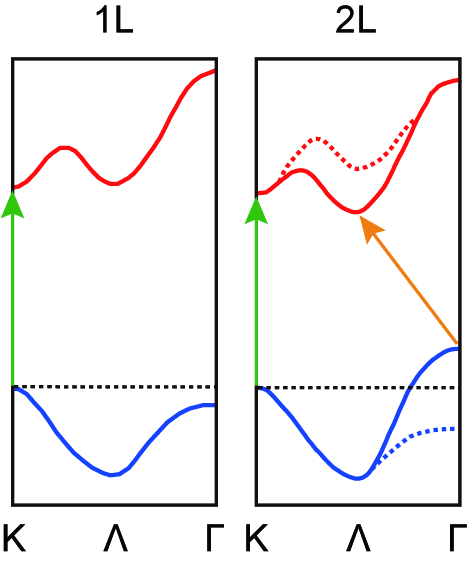


**Supplementary Figure 3. Schematic illustration of relevant band structures close to the band edges of monolayer and bilayer MoS_2_.** The green and orange arrows indicate the direct and indirect gap transitions, respectively. In bilayer MoS_2_, the conduction band at Λ point and valence band at Γ point split into two, but they are both doubly degenerate at K point, at which the direct gap transition occurs.





**Supplementary Figure 4. Asymmetry of the exciton lineshape in few-layer BP.** The fitting parameter *η* as a function of layer number. *η* is extracted from the fitting of *E*_11_ peaks using Eq. (3) of the main text, describing the asymmetric broadening by exciton localization. Error bars are from sample-to-sample variations.

**List of symbols for Notes 1 and 2:**

*ω*: the light frequency

*ħ*: the reduced Planck constant

*e*: the electron charge

*c*: the speed of light

*ε*_0_: the vacuum permittivity

*m*_0_: the free electron mass

*μ*: reduced effective mass, defined as 1/*μ* = 1/*m_e_* + 1/*m_h_*

*n_s_*: the refractive index

*A_r_*: the area of the 2D material

*E*_g_: bandgap

*E*_b_: exciton binding energy

*j*: subband index

*n*: exciton Rydberg series order

*χ*: the 2D sheet polarizability

*g_s_*(*g_v_*): the degrees of spin(valley) degeneracy

*α*: fine structure constant (~1/137)

*σ*_0_: the universal conductivity for single-layer graphene

*A*(*ħω*): the dimensionless absorption

*N*: layer number

**Supplementary Note 1. Exciton absorption in 2D semiconductors**

For 2D direct bandgap semiconductors, the dimensionless absorption of 2D bound (exciton) and unbound (continuum) states associated with one pair of conduction and valence 2D bands can be expressed as[^1^](#_ENREF_1) (for simplicity, the finite linewidth due to scatterings is not included)

(S1)

(S2)

where , $A_{0}=\frac{\pi e^{2}\left|P_{cv}\right|^{2}}{n_{s}c\varepsilon_{0}\omega m_{0}^{2}}$, is the Rydberg parameter for excitons, is the exciton Bohr radius, *P_cv_* is the momentum matrix element, Θ(*ε*) is the step function originates from the 2D joint density of states (DOS). Note that the spin degree of freedom of 2 is already included in Eqs. (S1) and (S2).

For the continuum state, the absorption is nearly a constant with slightly frequency (*ω*) dependence (see Supplementary Note 2), thus we have

$\frac{A_{0}}{2\pi a_{0}^{2}R_{y}}=C\cdot\frac{E_{g}}{\omega}$ (S3)

where *C* denotes the constant value. Inserting (S3) into (S1), and consider only the *s*-state (*n* = 1) excitons, it reads

(S4)

The integrated absorption (namely the area of the exciton peak) is

(S5)

Typically $R_{y}<<E_{g}$, thus

(S6)

In the 2D limit, $E_{b}^{2D}=4R_{y}$, thus

(S7)

It indicates that the integrated absorption (conductivity) is approximately proportional to the 2D exciton binding energy. This relation is also valid for other 2D quantum wells[^2^](#_ENREF_2) and provides an additional scheme to estimate 2D exciton binding energy.

For a few-layer BP, we can still treat it as a 2D system in the limit where the thickness is small compared to the exciton Bohr radius. Simple estimation based on the effective masses and dielectric constant of bulk BP gives a Bohr radius of ~5 nm[^3^](#_ENREF_3), [^4^](#_ENREF_4), which validates the approximation in our case.

For a *N*-layer BP supported on a PDMS substrate, $E_{b}^{2D}=\frac{3}{4\pi(\chi_{0}+N\chi_{1})}$, with *χ*_0_ = 6.5 Å and *χ*_1_ = 4.5 Å describing the dielectric screening effect contributed from the PDMS substrate and single-layer BP, respectively[^5^](#_ENREF_5). Thus, we have

$\sigma_{I}\propto\frac{1}{\chi_{0}+N\chi_{1}}$ (S8)

**Supplementary Note 2. Continuum absorption in 2D semiconductors**

Starting from the Fermi’s golden rule, for linearly polarized light (*ħω*) along $\overrightarrow{e}_{0}$, the dimensionless absorption *A*(*ħω*) of a free-standing 2D system can be expressed as (in SI units)[^1^](#_ENREF_1)

(S9)

where *n_s_* = 1 is the refractive index in vacuum, the momentum matrix element is defined as $\overrightarrow{P}_{cv}(\overrightarrow{k})=\langle c,\overrightarrow{k}|\overrightarrow{e}_{0}\cdot\overrightarrow{P}|v,\overrightarrow{k}\rangle$, and $E_{cv}(\overrightarrow{k})=E_{c\overrightarrow{k}}-E_{v\overrightarrow{k}}$, $\overrightarrow{k}$ is the wave vector. The sum of $\overrightarrow{k}$ is replaced by the following integral:

(S10)

Eq. (S10) can be converted to Eq. (S11) with the fine structure constant , and the degrees of spin and valley degeneracy of *g_s_* and *g_v_* taken into account.

(S11)

In the polar coordinate system, , and $dk=dE_{cv}\left(\frac{dE_{cv}}{dk}\right)^{-1}$, thus

(S12)

In the $\overrightarrow{k}\cdot\overrightarrow{p}$ model[^1^](#_ENREF_1), the effective Hamiltonian for the Bloch wavefunction $u_{n\overrightarrow{k}}$ is given by

(S13)

The subscript *x* (*y*) denotes the AC (ZZ) direction. For small values of $\overrightarrow{k}$, the eigenvalues are given by

, (S14)

$E_{g}\equiv E_{c}(0)-E_{v}(0)$, and the reduced effective mass $1/\mu_{x(y)}\equiv1/m_{ex(y)}+1/m_{hx(y)}$, thus

(S15)

On the other side, using two-band $\overrightarrow{k}\cdot\overrightarrow{p}$ model, it is obtained that

$\frac{1}{\mu_{x}}=\frac{4\left|\overrightarrow{P}_{cv}\right|^{2}}{m_{0}^{2}E_{g}}$ (S16)

Inserting (S15) and (S16) into (S12), we obtain

(S17)

For few-layer BP, *g_s_* = 2, *g_v_* = 1 (Ref.[^6^](#_ENREF_6)). Thus, near the band edge (*ħω* ≈ *E*_g_), we have  (or equals to the optical conductivity $\sigma_{0}\sqrt{\frac{\mu_{y}}{\mu_{x}}}$). The prefactor $\sqrt{\frac{\mu_{y}}{\mu_{x}}}$ originates from the band anisotropy, given that DOS $\propto\sqrt{\mu_{x}\mu_{y}}$, and $\overrightarrow{P}_{cv}(\overrightarrow{k})\propto1/\mu_{x}$. When *μ_x_* = *μ_y_*, it collapses to the isotropic case[^7^](#_ENREF_7). When it is on a substrate with refractive index *n_s_*, the absorption is modified to due to the local field correction.

Besides the common case for typical semiconductors discussed above, Stauber et al. proposed a generalized formula for optical conductivity in isotropic 2D systems[^7^](#_ENREF_7): *σ* = *g_s_*·*g_v_*·*ν*·*σ*_min_, where *σ*_min_ = *σ*_0_/4 is a minimal conductivity defined by the authors, equal to 1/4 of the universal optical conductivity of graphene, *ν* is related to the band curvature (*ε*_c,v_ ~ |*k*|*^ν^*, *ν* = 2 for parabolic dispersion, and *ν* = 1 for linear dispersion). Now, we consider three cases to check its validity, as summarized in Supplementary Table 1. Note that for massive Dirac fermions, the value is twice of *σ*_0_, due to the valley degeneracy.

Supplementary Table 1. Optical conductivity in different 2D cases

| Type of carriers | Examples | *g_s_* | *g_v_* | *ν* | Band anisotropy | *σ* |
| --- | --- | --- | --- | --- | --- | --- |
| Massive Schrodinger fermions | Few-layer BP | 2 | 1 | 2 | $\sqrt{\frac{\mu_{y}}{\mu_{x}}}$ | $\sigma=\sigma_{0}\sqrt{\frac{\mu_{y}}{\mu_{x}}}$ (*ħω* ≈ *E*_g_) |
| Massive Dirac fermions | Massive graphene | 2 | 2 | 2 | 1 | 2*σ*_0_ (*ħω* ≈ *E*_g_) |
| Massless Dirac fermions | K-doped few-layer BP[^8^](#_ENREF_8) | 2 | 2 | 1 | $\sqrt{\frac{\mu_{y}}{\mu_{x}}}$ | $\sigma=\sigma_{0}\sqrt{\frac{\mu_{y}}{\mu_{x}}}$ |

**Supplementary Note 3. Exciton absorption in MoS_2_**

Supplementary Fig. 2 shows the reflectance contrast (ΔR/R_0_) spectra of 1-4L MoS_2_, the two prominent peaks are assigned to A/B excitons, corresponding to direct-gap transitions at Κ point of the 2D Brillouin zone. It clearly shows that ΔR/R_0_ (proportional to the optical conductivity) increases with layer number, in sharp contrast to BP. This can be well understood from the band structure[^9^](#_ENREF_9). For clarity, Supplementary Fig. 3 illustrates the band structures of monolayer and bilayer MoS_2_. In monolayer, the conduction band minimum (CBM) and valence band maximum (VBM) both locate at K point. While in bilayer, CBM and VBM shift to Λ and Γ point, respectively, resulting in a direct-to-indirect gap transition. However, the dominant optical absorption features (A and B excitons) still come from the direct-gap transitions at Κ point, at which the conduction and valence bands do not split even with layer-layer interaction[^9^](#_ENREF_9). As a consequence, the joint DOS is doubly degenerate at K point in bilayer. Similarly, it is *N*-fold degenerate in *N*-layer. Therefore thicker MoS_2_ absorbs more light, as demonstrated in Supplementary Fig. 2.

**Supplementary References**

1. Chuang, S. L. *Physics of Photonic Devices* (John Wiley & Sons, Inc., Hoboken, New Jersey, 2009).

2. Feldmann, J. et al. Linewidth dependence of radiative exciton lifetimes in quantum wells. *Phys. Rev. Lett.* **59**, 2337 (1987).

3. Tran, V., Soklaski, R., Liang, Y. & Yang, L. Layer-controlled band gap and anisotropic excitons in few-layer black phosphorus. *Phys. Rev. B* **89**, 235319 (2014).

4. Asahina, H. & Morita, A. Band structure and optical properties of black phosphorus. *J. Phys. C: Solid State Phys.* **17**, 1839 (1984).

5. Zhang, G. et al. Determination of layer-dependent exciton binding energies in few-layer black phosphorus. *Sci. Adv.* **4**, eaap9977 (2018).

6. Low, T. et al. Tunable optical properties of multilayer black phosphorus thin films. *Phys. Rev. B* **90**, 075434 (2014).

7. Stauber, T., Noriega-Pérez, D. & Schliemann, J. Universal absorption of two-dimensional systems. *Phys. Rev. B* **91**, 115407 (2015).

8. Baik, S. S., Kim, K. S., Yi, Y. & Choi, H. J. Emergence of two-dimensional massless Dirac fermions, chiral pseudospins, and Berry's phase in potassium doped few-layer black phosphorus. *Nano Lett.* **15**, 7788 (2015).

9. Splendiani, A. et al. Emerging photoluminescence in monolayer MoS_2_. *Nano Lett.* **10**, 1271 (2010).
